# Applying bimolecular fluorescence complementation to screen and purify aquaporin protein:protein complexes

**DOI:** 10.1002/pro.3046

**Published:** 2016-09-26

**Authors:** Jennie Sjöhamn, Petra Båth, Richard Neutze, Kristina Hedfalk

**Affiliations:** ^1^ Department of Chemistry and Molecular BiologyUniversity of GothenburgGöteborgSE‐405 30Sweden

**Keywords:** aquaporin, calmodulin, YFP, BiFC, protein‐protein interaction, membrane protein complex, *Saccharomyces cerevisiae*

## Abstract

Protein:protein interactions play key functional roles in the molecular machinery of the cell. A major challenge for structural biology is to gain high‐resolution structural insight into how membrane protein function is regulated by protein:protein interactions. To this end we present a method to express, detect, and purify stable membrane protein complexes that are suitable for further structural characterization. Our approach utilizes bimolecular fluorescence complementation (BiFC), whereby each protein of an interaction pair is fused to nonfluorescent fragments of yellow fluorescent protein (YFP) that combine and mature as the complex is formed. YFP thus facilitates the visualization of protein:protein interactions *in vivo,* stabilizes the assembled complex, and provides a fluorescent marker during purification. This technique is validated by observing the formation of stable homotetramers of human aquaporin 0 (AQP0). The method's broader applicability is demonstrated by visualizing the interactions of AQP0 and human aquaporin 1 (AQP1) with the cytoplasmic regulatory protein calmodulin (CaM). The dependence of the AQP0‐CaM complex on the AQP0 C‐terminus is also demonstrated since the C‐terminal truncated construct provides a negative control. This screening approach may therefore facilitate the production and purification of membrane protein:protein complexes for later structural studies by X‐ray crystallography or single particle electron microscopy.

## Introduction

Membrane protein structural biology has advanced dramatically over the last decade.^1^ The field is now at a stage where targets can be designed for protein production and structural analysis.^2^ It has become almost routine to achieve high‐resolution structures using X‐ray crystallography and single particle electron microscopy^3^ has shown rapid advances over recent years. A major challenge in achieving a deeper understanding of membrane protein function within the cell, however, is to develop tools that allow researchers to achieve structural insight into the mechanisms governing communication between proteins. Protein‐protein interactions (PPIs) are fundamental to all biological processes such as physical motion, metabolism and signalling cascades and high resolution structures that reveal the details of these interactions are expected to strongly influence the progression of the field.^4^

Most methods for screening and verifying PPIs are developed for soluble proteins but many can, with adaptions, be applied to integral membrane proteins.^5^, ^6^ A major shortcoming in several well‐established methods is a tendency to over interpret results, which gives rise to a large fraction of false positives.^7^ Both the two‐hybrid screening^8^ and co‐precipitation,^9^, ^10^ for example, normally require verification of the PPI results *in vivo*.^7^ Structural information on PPI complexes would both provide compelling evidence of the protein:protein interaction while delivering additional new insights, particularly concerning the flexible hydrophilic domains which typically play a vital part in the actual interaction. In order to make progress in the field of membrane protein:protein interactions there is therefore a need to develop generic strategies for the production and purification of stable complexes, especially for eukaryotic targets, as has previously been developed for the production of individual membrane protein targets.^11^

Calmodulin (CaM) is an important regulator of many membrane proteins including several human aquaporins. In the case of mammalian aquaporin 1 (AQP1), the water channel can be regulated by translocation to the plasma membrane and this occurs within 30 s once it is activated by CaM.^12^ Another example is provided by Aquaporin 6 (AQP6), which is an anion channel that is primarily produced in the kidney where the binding of CaM is important for its functional role. A putative CaM binding site has been located to the N‐terminus of AQP6.^13^ A well‐studied third example is the interaction between aquaporin 0 (AQP0) and CaM. Analysis by NMR spectroscopy^14^ and electron microscopy (EM)^15^ combined with biochemical analysis and modelling show that CaM inhibits water permeation through AQP0 by binding to its C‐terminus (residue 223–242) and thereby inducing the structural occluding of the water channel. Two CaM molecules bind to the AQP0 tetramer in the presence of calcium^14^ and the binding of CaM to the C‐terminus of AQP0 can be regulated by phosphorylation, whereby phosphorylation decreases the affinity of AQP0 for CaM 20‐ to 50‐fold.^16^ The inhibition of water flow upon CaM binding is suggested to work by cooperativity between adjacent subunits in an allosteric fashion^17^ and a point mutation which causes polymorphic congenital cataract (R233K) gives rise to a weaker interaction with CaM.^18^

Since a deeper understanding of the mechanism of CaM regulation of aquaporins requires high resolution structures, complexes provide attractive PPIs for technological developments in this direction. In this work we therefore use these AQP‐CaM interactions to develop a screening approach that ensures that the PPI complex forms *in vivo* and remains intact during membrane extraction and purification. Moreover, we aimed to incorporate fluorescence as a readout since this increases the sensitivity of the screen, allows membrane localization to be established using confocal microscopy,^19^ and fluorescence markers have been of great value during protein purification since the target can be followed and conditions optimized using fluorescent size exclusion chromatography (FSEC).^20^ This is particularly helpful for membrane proteins since detergents suitable for crystallization can be screened and mutants evaluated at an early stage in the process.^21^, ^22^ This rational leads to the application of bimolecular fluorescence complementation (BiFC) as an interesting technology since the fluorescent protein serves two purposes: (i) it anchors weak PPIs that are otherwise transient in nature; and (ii) it provides a convenient signal for detection since fluorescence is applied.^23^

**Figure 1:**
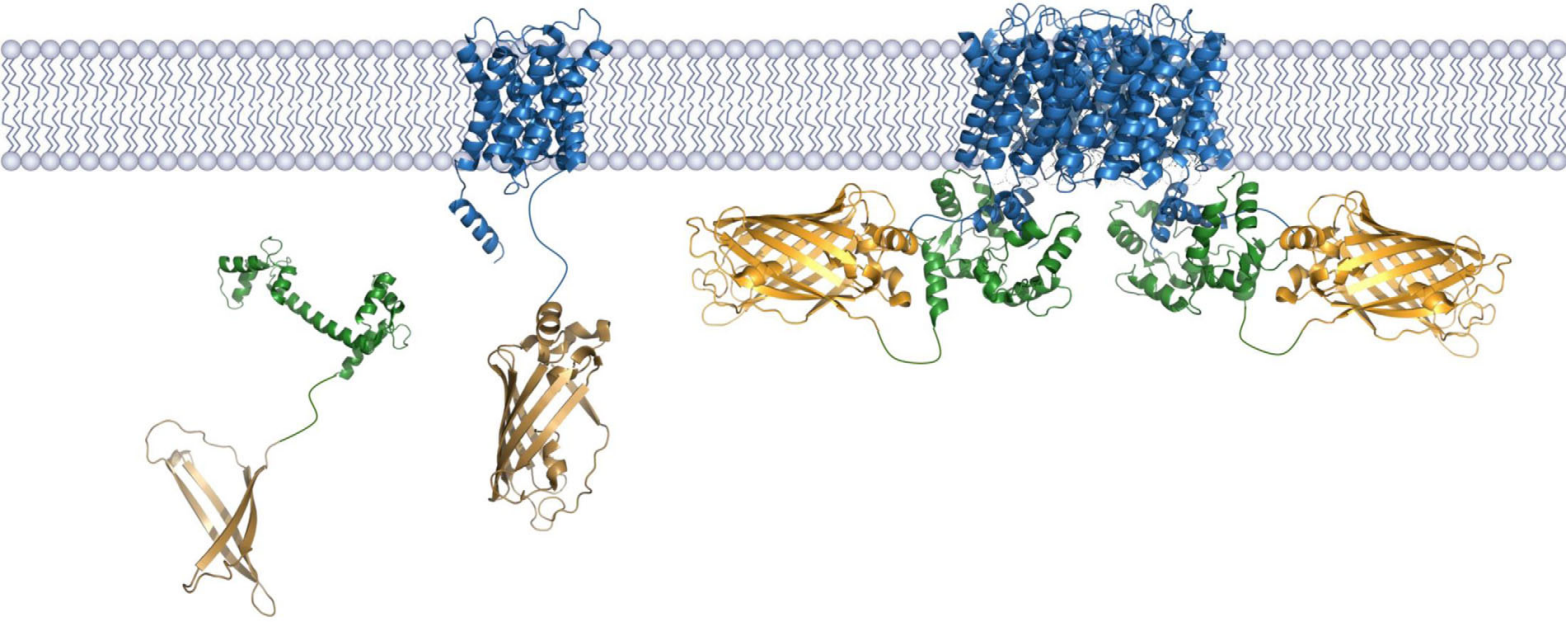
Schematic representation of the AQP0‐CaM complex connected by BiFC. In this representation, two CaM bind to the AQP0 tetramer whereupon the YFP fragments reassemble, mature and fluoresce.

BiFC is a protein complementation assay whereby a fluorescent protein (usually YFP) is divided into two nonfluorescent fragments, each of which is fused to the two proteins of interest that are believed to form a PPI (Fig. 1). These complementary fusions are produced in a suitable host and, as the targets interact, the two halves of the YFP assemble and mature *in vivo*. The fluorescence signal can be used to assess the localization and stability of the protein complex or to estimate the yield. BiFC is a flexible approach that has been used to detect PPIs in plants,^24^ *in vivo* interactions in *Saccharomyces cerevisiae*,^25^ screening for protein‐protein interactions in human cells^26^ and to investigate heterotetramerization of aquaporins in *Arabidopsis thaliana*.^27^ Since the assembly of the two YFP fragments is almost irreversible,^28^ this results in a stable PPI complex that can be monitored by fluorescence. We demonstrate this technology using AQP0‐CaM as a model complex and thereby validate the AQP0‐CaM interaction, we show that the presence of the C‐terminus is needed for the interaction to take place, and we demonstrate how fluorescence can be used to follow the complex throughout protein purification. We then extend the scope of this study to include the AQP1‐CaM interaction, the AQP6‐CaM interaction, and demonstrate an interaction between AQP0 and AQP2. These results suggest that the scope of potential target complexes can easily be expanded to include other human aquaporins with established interaction partners^7^ or be applied very generally for validating and purifying PPIs for any membrane protein of choice.

## Results

### Aquaporin tetramerization *in vivo*

**Figure 2:**
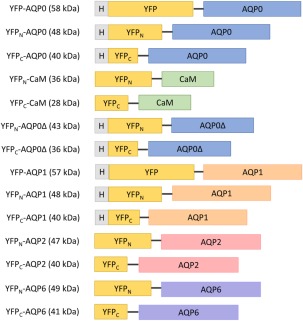
Schematic showing all fusion constructs used in this study. Full length YFP, the N‐terminal part (YFP_N_) and the C‐terminal part (YFP_C_) of YFP were fused to the N‐termini of AQP0, AQP1, AQP2, AQP6, or CaM. The expected molecular weight for the monomeric form of each protein product is given in parenthesis. H represents the 6x‐His purification tag.

**Figure 3:**
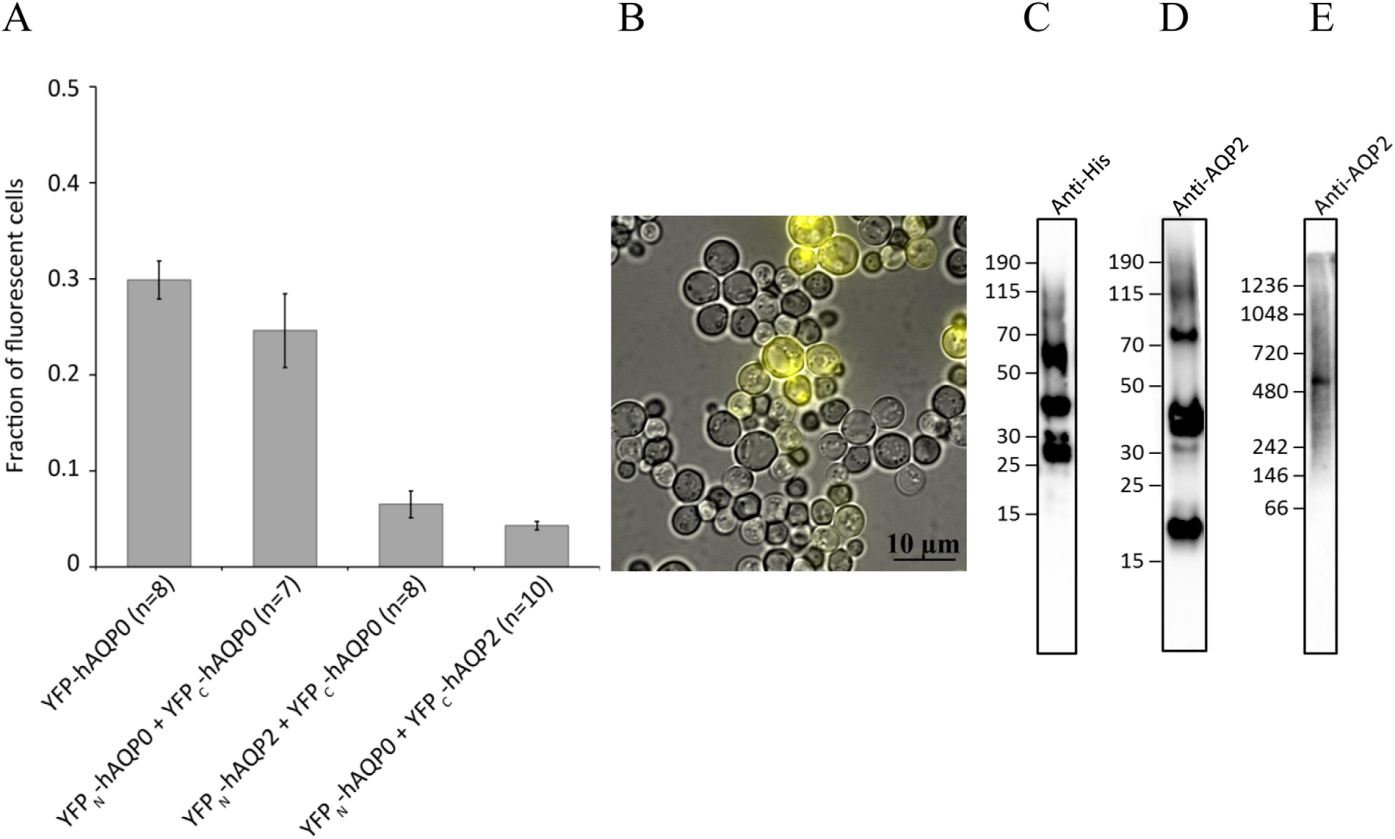
BiFC analysis of AQP‐AQP complexes. (A) The fraction of fluorescent cells producing AQP0 tetramers linked by BiFC (YFP_N_‐AQP0 + YFP_C_‐AQP0) is shown and compared to YFP‐AQP0, which is used as a positive control, showing fluorescence of full‐length YFP fused to AQP0. The interaction of AQP0 and AQP2 provides a reference complex for which two BiFC pairs are tested in the fusion design, see Figure 2. The fraction of fluorescent cells is based on 7 to 10 separate transformations of each construct. (B) A typical microscopy image of cells producing the positive control YFP‐AQP0. (C) Immunoblot analysis (SDS‐PAGE) of the AQP0‐AQP2 reference complex (YFP_N_‐AQP0 + YFP_C_‐AQP2) purified by Ni‐NTA chromatography using the anti‐his antibody high‐lighting the YFP_N_‐AQP0 part of the complex (∼48 kDa), or (D), the anti‐AQP2 antibody high‐lighting the YFP_C_‐AQP2 part of the complex (∼40 kDa) in the same fraction. (E) Immunoblot analysis (Native‐PAGE) of the AQP0‐AQP2 reference complex after Ni‐NTA purification using the anti‐AQP2 antibody high‐lighting a possible complex in various oligomeric forms.

All YFP‐AQP0 fusion constructs designed for this study consist of the full‐length YFP, the N‐terminal part of YFP (YFP_N_, residues 1–154) or the C‐terminal part of YFP (YFP_C_, residues 155–238) expressed together with the protein of interest (Fig. 2). Since the YFP‐AQP0 construct does not require a protein interaction to yield fluorescence, it represents the maximum fluorescence obtainable in our studies and was therefore used as a positive control. Fluorescence microscopy images indicate fluorescence from YFP in the membrane, but also spread throughout the cytoplasm, indicating that not all of the produced protein is properly localized [Fig. 3(B)]. This effect is common in membrane protein overproduction, whether labeled with GFP or not, because the translocation machinery can get overloaded when membrane protein targets are expressed from strong promoters.^29^

BiFC in *S. cerevisiae* was validated by observing the formation of AQP0 tetramers using the YFP_N_‐AQP0 + YFP_C_‐AQP0 constructs. The fraction of fluorescent cells for this combination was similar to YFP‐AQP0, indicating that AQP0 spontaneously formed tetramers when the BiFC fragments were present. As a reference complex, we also evaluated the complex formation of AQP0 and AQP2 using BiFC. These aquaporin homologues, sharing 60% sequence identity, are distributed in different human tissues. Two combinations of constructs were evaluated for this analysis by swapping which half of YFP was fused to AQP0 or AQP2, resulting in YFP_N_‐AQP0 + YFP_C_‐AQP2 and YFP_N_‐AQP2 + YFP_C_‐AQP0 combinations, respectively (Fig. 2). Both combinations gave rise to low fluorescence: the YFP_N_‐AQP0 + YFP_C_‐AQP2 combination showed some residual fluorescence whereas the YFP_N_‐AQP2 + YFP_C_‐AQP0 combination showed even lower levels of fluorescence (Fig. 3).hAQP0 and hAQP2 are distributed in the eye and kidney respectively and are not expected to have any functional reason to form heterotetramers. Applying the BiFC assay to these targets (YFP_N_‐AQP2 + YFP_C_‐AQP0 and YFP_N_‐AQP0 + YFP_C_‐AQP2) gave rise to fluorescence in yeast, however, and we therefore evaluated this result further. To this end, we first purified the AQP0‐AQP2 complex (YFP_N_‐AQP0 + YFP_C_‐AQP2) using Ni‐NTA chromatography and his‐tag fused to the AQP0 part of the BiFC complex. The fraction to elute from the column was analyzed by SDS‐PAGE and immunoblot, where both anti‐his and anti‐AQP2 antibodies were used to analyse the same sample [Fig. 3(C,D)]. From immunoblot staining both the YFP_N_‐AQP0 (∼48 kDa) and YFP_C_‐AQP2 (∼40 kDa) components of the complex could be detected, which establishes that AQP2 co‐purifies with AQP0 when bound in a BiFC complex. Since BiFC complexes frequently degrade when denatured in SDS, which is also seen in this study, we further analyzed the AQP0‐AQP2 complex on an immunoblot based on a Native‐PAGE [Fig. 3(E)]. This immunoblot shows several bands above 242 kDa, detected by the anti‐AQP2 antibody, which could be in agreement with oligomeric forms of the AQP0‐AQP2 BiFC complex. In conclusion, our data support the idea that AQP0 and AQP2 form a BiFC complex, but whether this is due to heterotetramerization or due to the presence of mixed tetramers in the octameric complex, we cannot establish. The possibility of spontaneous heterotetramization of human aquaporins, as exemplified by AQP0 and AQP2 in this study, should at least be acknowledged since heterotetramers have been reported for plant aquaporins,^27^, ^30^ where heterotetramers of various TIPs and MIPs have been reported for *Arabidopsis thaliana* aquaporins when produced in *S. cerevisiae*.^27^ In addition, human aquaporins have shown to regulate each other, as shown for AQP2 and AQP5.^31^

### AQP‐CaM interaction *in vivo*

**Figure 4:**
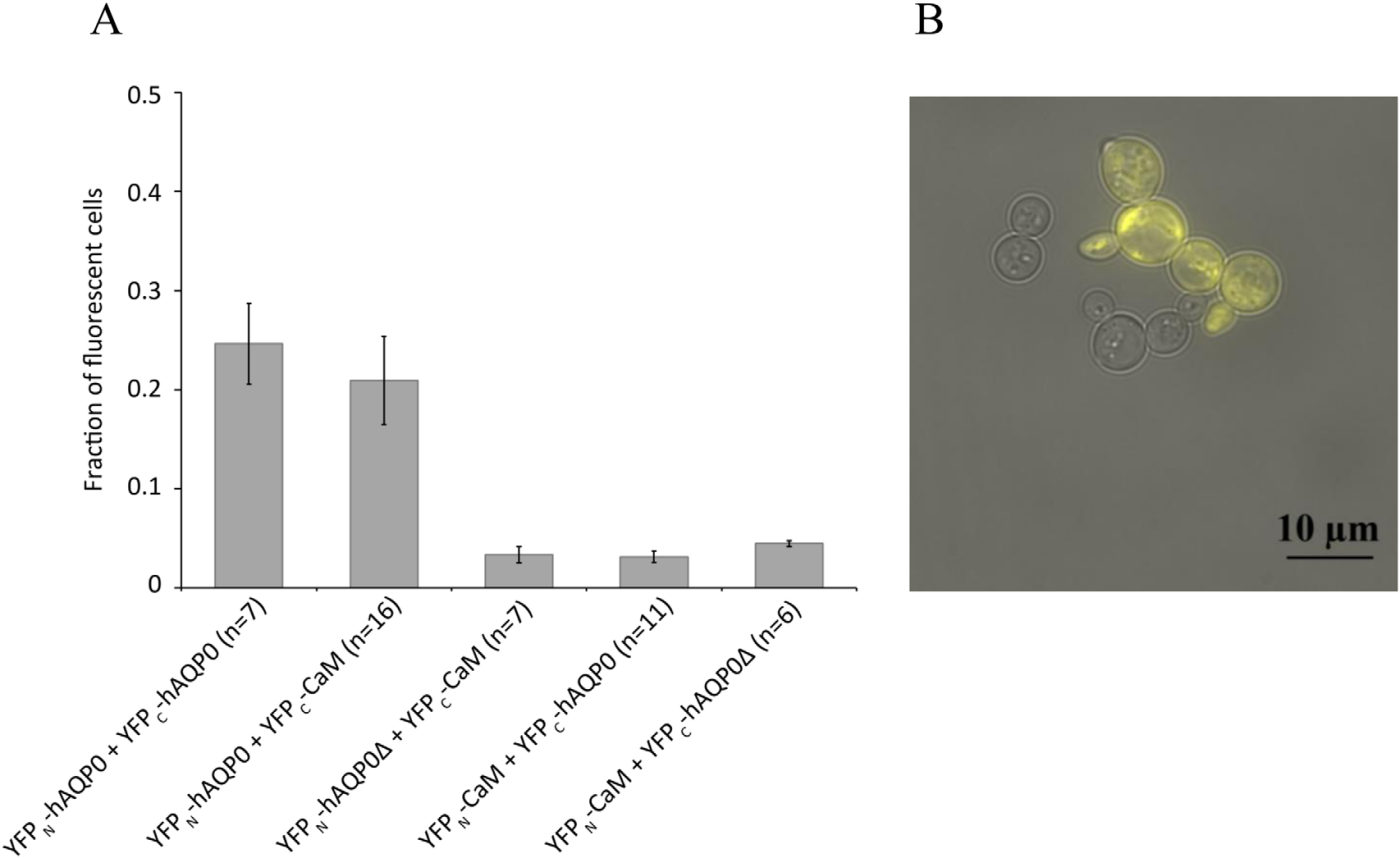
BiFC analysis of the AQP0‐CaM complex. (A) Fraction of fluorescent cells producing two variants of the AQP0‐CaM BiFC complex compared to AQP0 tetramers linked by BiFC. Removing the CaM interacting domain in the AQP0 C‐terminus (hAQP0Δ) significantly reduces the fraction of fluorescing cells when tested for the functional BiFC pair (YFP_N_‐AQP0 + YFP_C_‐CaM). The fraction of fluorescent cells is based on 6 to 16 separate transformations of each construct. (B) A typical microscopy image of yeast cells producing the successful AQP0‐CaM BiFC complex (YFP_N_‐AQP0 + YFP_C_‐CaM).

We next validated the interaction between AQP0 and CaM using BiFC and compared these results against the BiFC results from the AQP0 tetramerization. AQP0 was fused to the N‐terminal half of YFP and CaM to the C‐terminal half of YFP (YFP_N_‐AQP0 + YFP_C_‐CaM, Fig. 2). The resulting fluorescent signal was of a similar level to that of the AQP0 tetramer [Fig. 4(A)], which is formed when recombinantly produced in *S. cerevisiae*, showing that a physiologically active AQP0‐CaM complex is also formed under these conditions. Furthermore, the high fluorescence yields of the YFP_N_‐AQP0 + YFP_C_‐CaM confirms the stability of the YFP once the complex has assembled. Fluorescence microscopy images indicates that the AQP0‐CaM complex is partly localized to the membrane [Fig. 4(B)] although, as with the AQP0 constructs above, quite some fluorescence is also observed elsewhere in these cells. These levels of observed fluorescence were dramatically reduced by truncating the AQP0 C‐terminus [removal of residue 221–271; Fig. 4(A)]. These results support the hypothesis that the AQP0 C‐terminus contains the CaM binding site and concurs with the C‐terminal being important for proper trafficking.^32^

In contrast with the YFP_N_‐AQP0 + YFP_C_‐CaM fusion construct, the combination of the N‐terminal half of YFP to CaM and the C‐terminal half of YFP to AQP0 (the YFP_N_‐CaM + YFP_C_‐AQP0 fusion construct) did not give rise to a fluorescent signal, implying that the AQP0‐CaM complex was not formed with this combination of fusions. An absence of a fluorescent signal for this unproductive BiFC pair was also true when the C‐terminal binding domain for CaM was deleted [Fig. 4(A)]. We therefore speculate that steric hindrances may make one of the fusion constructs viable with BiFC whereas its complementary construct is not. Taken together, these results illustrate the need for testing alternative fusion constructs when evaluating the BiFC approach when targeting a specific complex formation.

**Figure 5:**
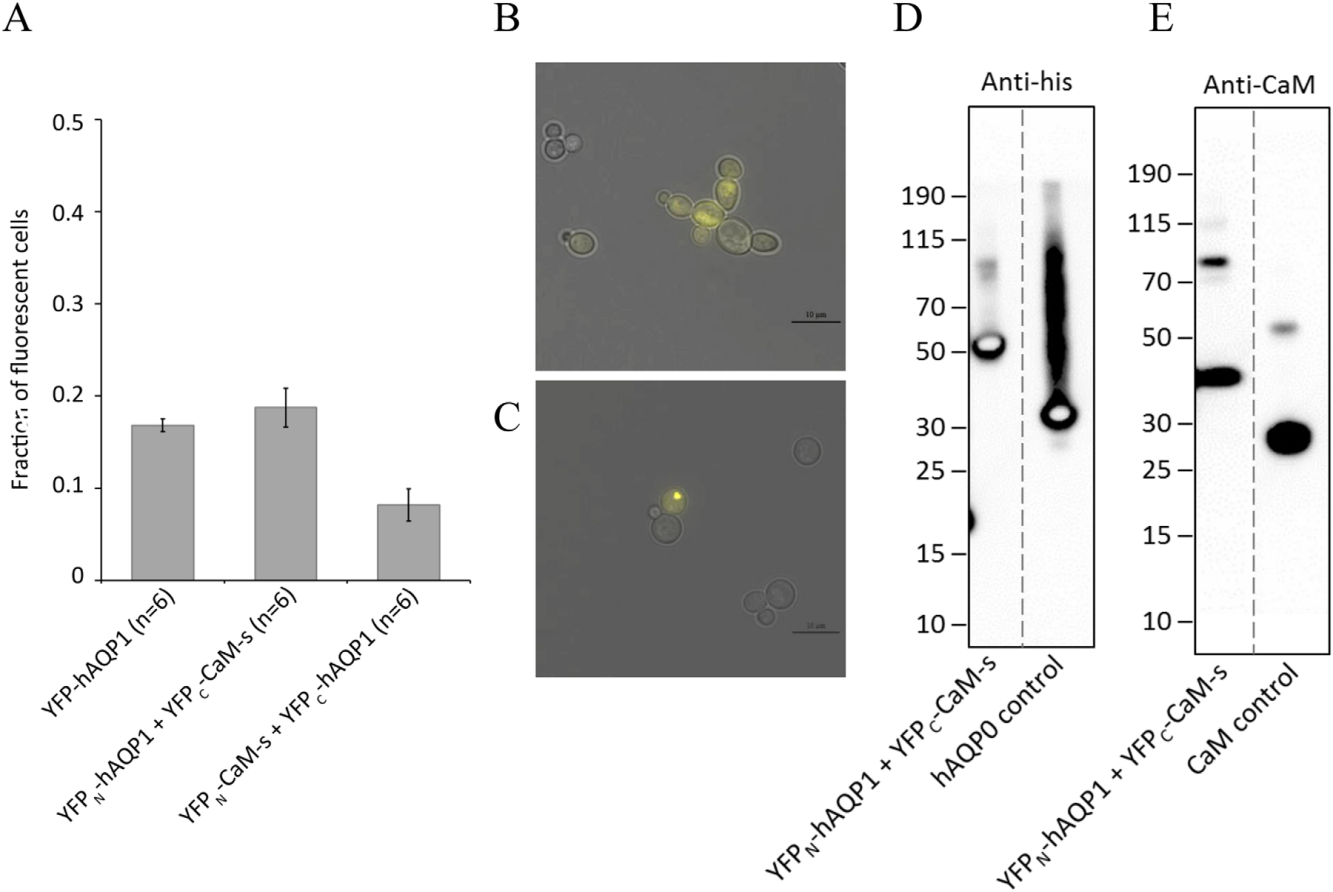
BiFC analysis of the AQP1‐CaM (YFP_N_‐AQP1 + YFP_C_‐CaM‐s) and AQP6‐CaM (YFP_N_‐AQP6 + YFP_C_‐CaM‐s) complexes. (A) The fraction of fluorescent cells producing the hAQP1‐CaM BiFC complex where two fusion variants were tested and compared to YFP‐hAQP1, which was used as a positive control, showing fluorescence of full‐length YFP fused to AQP1. The fraction of fluorescent cells is based on six separate transformations of each construct. (B) A typical microscopy image showing cells producing the successful hAQP1‐CaM BiFC complex (YFP_N_‐AQP1 + YFP_C_‐CaM‐s). (C) A typical microscopy image showing a low fluorescent signal for AQP6, the positive control (YFP‐AQP6), which is mainly localized to intracellular aggregates. (D) Immunoblot analysis (SDS‐PAGE) of the AQP1‐CaM complex (YFP_N_‐AQP1 + YFP_C_‐CaM‐s) purified by FSEC, using the anti‐his antibody, showing a dominant band of 48 kDa corresponding to the AQP1 part of the complex (YFP_N_‐AQP1), or (E) the anti‐CaM antibody, showing a dominant band of 28 kDa corresponding to the CaM part of the complex (YFP_C_‐CaM‐s). On both immunoblots, a band at about 76 kDa could be observed, possibly corresponding to the full AQP1‐CaM complex (YFP_N_‐AQP1 + YFP_C_‐CaM‐s) in its monomeric form.

To further evaluate the generality of our method for screening and purification of membrane protein complexes we included hAQP1‐CaM and hAQP6‐CaM as additional targets within this study. As above, we used the full fusion with YFP as a positive control (YFP‐AQP1 and YFP‐AQP6) and we cloned several variants of the constructs in our screen for fluorescence (Fig. 2). One of the BiFC pairs (YFP_N_‐AQP1 + YFP_C_‐CaM‐s) gave rise to fluorescence of the same order of magnitude as the positive control, whereas the other construct (YFP_N_‐CaM‐s + YFP_C_‐AQP1) did not [Fig. 5(A)], similar to the results obtained for the YFP0‐CaM complex. Our analysis of a putative BiFC complex of hAQP6 and CaM, however, resulted in very few fluorescent cells (data not shown). We suspect that the underlying reason for this outcome is the relatively low production level of hAQP6 in yeast, which we previously showed when expressed in *Pichia pastoris*.^33^ The microscopy images of the YFP‐AQP6 in yeast confirm the production of intracellular aggregates of fluorescent protein within the cytosol [Fig. 5(C)]. The AQP6‐CaM BiFC complex gives similar results (data not shown).

Since YFP_N_‐AQP1 + YFP_C_‐CaM‐s successfully formed a BiFC complex, we purified the hAQP1‐CaM complex using ion exchange and SEC chromatography. The fluorescent peak was analyzed by SDS‐PAGE and immunoblot staining using anti‐his and anti‐CaM antibodies, respectively [Fig. 5(D,E)]. Both parts of the BiFC complex were detected, with an estimated mass for YFP_N_‐AQP1 of ∼48 kDa and YFP_C_‐CaM‐s of ∼28 kDa, as well as the monomeric from of the BiFC pair, YFP_N_‐AQP1 + YFP_C_‐CaM‐s of ∼76 kDa. We thereby conclude that hAQP1‐CaM forms a successful BiFC pair that can be screened and purified from *S. cerevisiae*.

### Larger scale production and purification of the AQP0‐CaM complex utilizing the YFP tag

Our results using fluorescence microscopy demonstrate that it is possible to co‐express two fusion proteins in *S. cerevisiae*. The resulting BiFC signal indicates the formation of a membrane protein:soluble protein complex which is directed for insertion into the membrane. For downstream structural studies it is also necessary to extract this complex from the membrane using detergents and to further purify the complex. This is not trivial since detergents supplant the biological membrane bilayer and are known to interfere with protein:protein interactions. For example, large integral membrane protein complexes frequently loose subunits when extracted from the membrane and crystallized in detergent.^34^ Purification trials and SEC analysis of isolated AQP0 show a single SEC peak corresponding to the size of the complex tetramer. In contrast, the AQP0 and the CaM proteins do not form a stable complex when expressed and purified separately and then mixed, even in the presence of Ca^2+^, with two separate peaks resulting when analyzed by SEC (Supporting Information Fig. S1). From these observations we conclude that the affinity between AQP0 and CaM is significantly lower than that between the AQP0 monomers.

Our use of BiFC for protein production enables YFP to help keep the AQP0‐CaM linked together during purification, as will it ensure stoichiometric ratios of the two proteins during crystallization. Cells expressing YFP_N_‐AQP0 + YFP_C_‐CaM were therefore grown in large scale and the AQP0‐CaM complex was purified. To assure isolation of a homogenous population of the desired complex, our initial purification trials tested a dual tag approach combining affinity purification based on his‐ and strep‐tag, respectively (data not shown). This approach, however, did not full‐fill our requirements of high‐yields of the authentic product since we found poor binding of the strep‐tag, high levels of degradation, and a low final yield. These disappointing outcomes were also observed when purifying the AQP0‐AQP2 complex using the same approach (data not shown).

**Figure 6:**
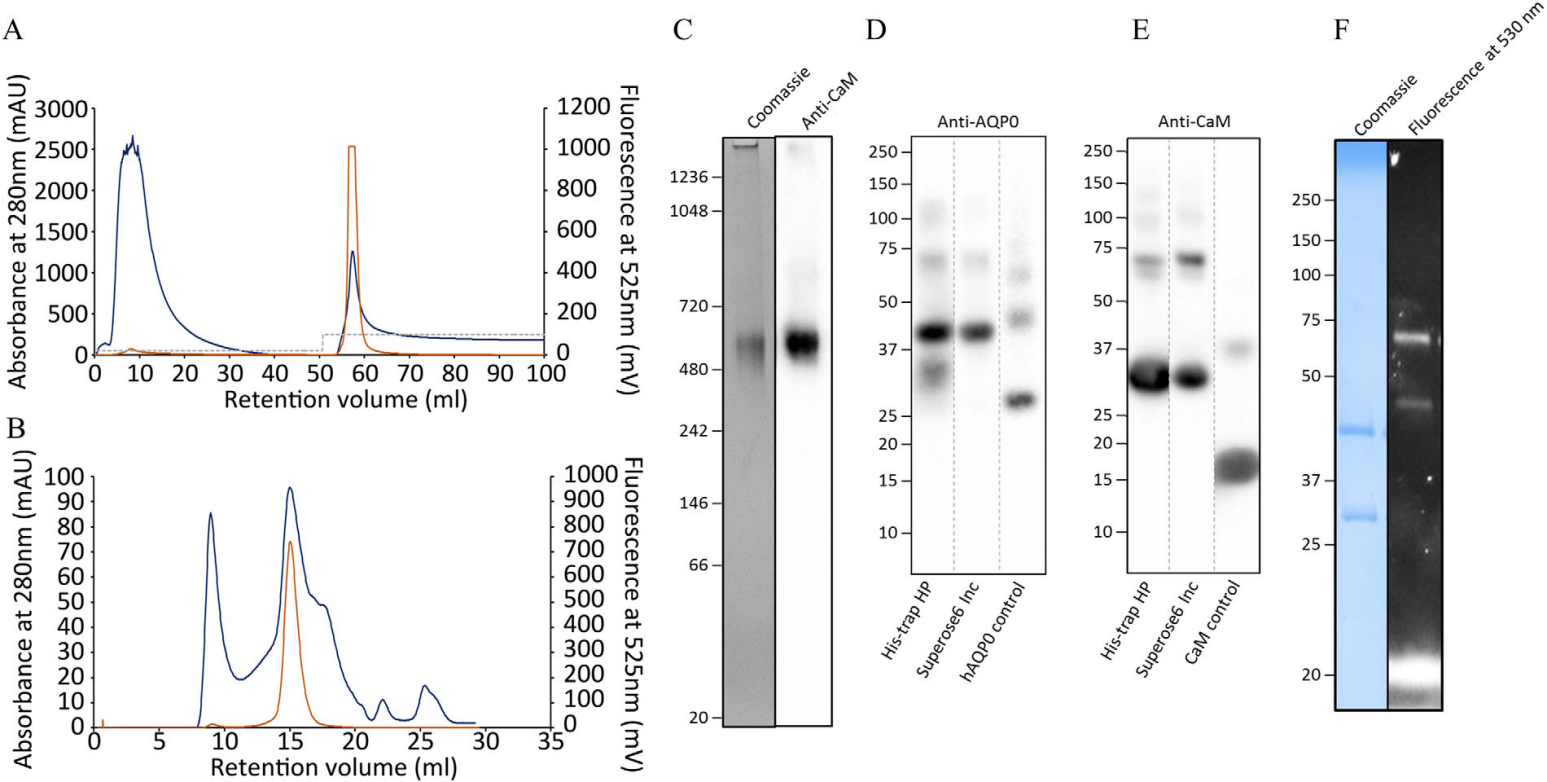
Purification of the AQP0‐CaM complex (YFP_N_‐AQP0 + YFP_C_‐CaM*). (A) The chromatogram from the Ni‐NTA purification (HisTrap HP column) showing a perfect overlap of the absorbance and the fluorescent peak. (B) The chromatogram from the FSEC (Superose6 Increase) purification showing a main protein peak correlating with the fluorescence at an elution volume corresponding to a molecular weight of approximately 500 kDa. (C) The fluorescent protein peak from FSEC was analyzed using Native‐PAGE and immunoblot using the anti‐CaM antibody. Only one band is observed indicating that one form of the complex is purified, corresponding to the size of an octameric form of the complex with four CaM and eight AQP linked by YFP. The FSEC peak was analyzed further by immunoblots based on SDS‐PAGE analysis (NuPAGE 4‐12% Bis‐tris gels, Thermofischer) using the anti‐AQP0 (D) and anti‐CaM antibody (E), respectively. For each immunoblot, the pure protein, AQP0 or CaM, was used as positive control. The immunoblot analyses show the individual parts of the AQP0‐CaM complex at the expected molecular weights (48 kDa for YFP_N_‐AQP0, D, and 29 kDa for his‐YFP_C_‐CaM, E). (F) SDS‐PAGE analysis and in gel‐fluorescence (Mini‐Protean TGX gels) of the FSEC peak showing fluorescent degradation products possibly corresponding to AQP0‐YFP (54 kDa) and CaM‐YFP (43 kDa).

We therefore purified the AQP0‐CaM BiFC (YFP_N_‐AQP0 + YFP_C_‐CaM*, where CaM* indicates that the His‐tag is on the CaM moiety) complex by combining Ni‐affinity chromatography with SEC. Our absorbance peak from Ni‐NTA purification shows a pure fraction with a reasonable yield where the absorbance‐ and fluorescent peaks perfectly overlap [Fig. 6(A)]. The yield of the purified complex was estimated to 2.8 mg from 40 g cells. Using FSEC and detection at 280 nm in the final step of the purification, the elution profile shows some protein aggregation eluted at 8 to 9 mL and a main absorbance peak overlapping with the homogenous fluorescent peak at 15 mL [Fig. 6(B)]. This is consistent with the molecular weight of the BiFC complex of AQP0 and CaM in its octameric form (∼496 kDa). Native‐PAGE analysis supports that the fluorescent peak at 15 mL contains protein with a size of approximately 500 kDa [Fig. 6(C)] which correlates to the molecular weight of the full complex (AQP0‐YFP‐CaM) assembled as an octamer. The propensity of AQP0 to form tetramers that further assemble into octamers is well known and reflects the dual function of AQP0 as a water transport protein and its involvement in forming junctions between adjacent cell membranes in the eye lens.^35^ Immunoblot analysis of the same peak with antibodies raised against CaM verified the presence of CaM at the size of the AQP0‐CaM complex (500 kDa) [Fig. 6(C)] confirming that it indeed is the intact complex that has been purified. The fluorescent peak from SEC was further analyzed by SDS‐PAGE with corresponding immunoblots and in‐gel fluorescence. From the Coomassie stained gel, we observe the individual parts of the BiFC complex, YFP_N_‐AQP0 (48 kDa) and his‐YFP_C_‐CaM (29 kDa), which is also confirmed by mass spectrometry analysis (data not shown). Those products were further verified on immunoblots using the anti‐hAQP0 and anti‐CaM antibodies, respectively [Fig. 6(D,E)], where also a monomeric from of the BiFC pair, YFP_N_‐AQP0 + YFP_C_‐CaM*, could be detected (76 kDa). A complementary SDS‐PAGE gel was performed where the same samples were analyzed for fluorescence. Two fluorescent bands with intact YFP parts were obtained, possibly connected to each moiety of the complex, AQP0 and CaM, corresponding to products of 54 kDa and 43 kDa, respectively [Fig. 6(F)].

In summary, by a two‐step procedure we purified a hAQP0‐CaM complex which most likely arises from an octameric state of the aquaporin (∼500 kDa). Immunoblot staining combined with mass‐spectrometry analysis confirmed the presence of AQP0 and CaM, supporting our interpretation that we have succeeded with a homogenous preparation of the desired target. From these results we estimate that we can purify, by detergent extraction and affinity chromatography, up to 0.6 mg of the AQP0‐CaM complex per litre of yeast culture. The sample is sufficiently homogenous after SEC purification that it should be suitable for further structural analysis including crystallization trials. Moreover, the size of the complex (∼500 kDa) and its eight fold‐symmetry suggest that this preparation may also be amenable for structural characterization using single particle cryo‐electron microscopy.^36^, ^37^

## Discussion

Membrane protein complexes are challenging but highly desired targets in structural biology. Obtaining homogenous samples of membrane protein complexes is problematic, especially for low affinity interaction partners for which the complex may dissociate during purification. In this work, we have investigated multiple strategies in order to establish a system that fulfils the requirement of acceptable background signal when screened in whole cells; high stability for the overproduced complex during purification; and sufficient yield of the final product for further structural characterization. We initially tried to build a system serving this purpose based upon an in‐house developed cell‐free expression system^38^ or using protein production in the yeast *Pichia pastoris*. We ultimately found, however, that *S. cerevisiae* provides the best system for which we can both screen and visualize our targets in whole cells and subsequently purify our complex with an acceptable yield.

While at first sight the entire procedure may appear somewhat extensive and laborious, the main benefit with the strategy presented here is that the whole process from screening to production can be performed in the same host. *S. cerevisiae* is also a very mature protein production host and this offers opportunities for optimization and is amenable to automation. The method developed here is based upon standard laboratory techniques which are well established, robust and can be set up in a multiple well format, which minimizes the time and effort spent on the first phase of the project. Fluorescence microscopy also facilitates early evaluation of the promise of the technique and in particular indicates if the desired product is being targeted to the membrane or being aggregated within the cell. For the AQP0‐CaM interaction, as well as the AQP1‐CaM complex, a substantial fraction of the expressed BiFC protein complex is localized to the membrane, which is indicated by the microscopy images and further supported by the membrane fractionation for purification purposes, and this supports the notion that the proteins are correctly folded and assembled and hence likely stable [Figs. 4(B) and 5(B)]. We therefore suggest that should the membrane localization fail, new constructs could be designed and evaluated for proper localization before a large amount of effort is committed to large scale production, purification and crystallization. It may also be possible to develop higher throughput approaches that are based on the fluorescent YFP BiFC readout using cell sorting approaches such as FACS, which may thereby allow automatization of another step in the process.

### BiFC helps maintain the PPI complex during purification

When comparing the results of purification of the BiFC AQP0‐CaM complex with the scenario where AQP0 and CaM were expressed independently and mixed, it is apparent that the AQP0‐CaM complex is more stable when using BiFC constructs. In particular for both cases where AQP0 and CaM were produced separately and mixed with and without Ca^2+^ being present, these proteins dissociated from each other during the SEC step, resulting in two peaks (Supporting Information Fig. S1). In contrast, our results from BiFC purification demonstrate that this technology provides a novel tool that helps to keep the complex together (Figs. 4 and 6), which is essential for further structural analysis. We expect that the purified complex contains two CaM bound to each AQP0 tetramer. This stoichiometry, however, is hard to evaluate from the SDS‐PAGE analysis [Fig. 6(F)] since the intensity of these two moieties when stained by Coomassie have similar intensities. Control measurements from samples of AQP0 and CaM indicate that the aquaporin is less efficiently stained relative to its soluble partner (data not shown), which potentially leads to misleading quantification from the Coomassie gel. Indeed, poor staining of aquaporins is known from the literature.^39^, ^40^

### Variation between transformations

Competent *S. cerevisiae* cells transformed with the BiFC constructs were analyzed for whole‐cell fluorescence and enabled AQP0‐CaM, AQP1‐CaM, AQP6‐CaM, and AQP0‐AQP2 interactions to be visualized. During these experiments, it was evident that some transformations which yielded colonies and behaved normally during culturing, deviated from the general pattern observed in the microscopy analysis. To account for possible variations in protein production between individual yeast colonies, transformations were performed in replicates of at least 10. This protocol allowed atypical behavior from a specific yeast transformation to be excluded and the statistical analysis for cell counting is based on 6 to 16 separate transformations of each construct (Figs. 3, 4, 5). Plasmid instability is one possible explanation for the observed variation between yeast transformants and in *S. cerevisiae,* plasmid stability can be an issue even when growing cells in selective media. When transformed with a single plasmid, plasmid losses ranges from 3% to 5% depending on the selective marker used^41^ which is expected to result in 10% to 30% of the final population lacking the plasmid. Another consideration is that the kinetics in the YFP reassembly and maturation is dependent on the affinity of the protein complex formed and is thought to be in the range from minutes to hours.^28^ Alterations to proteins that alter the PPI affinity may therefore change the rate of fluorescence build up. For these reasons, it is of major importance to define a growth procedure and harvest time in order to minimize the variation in fluorescence between different samples.

### Construct design may be critical for constructive BiFC pair formation

In BiFC, the way the fluorophore fragment and the protein of interest are combined clearly plays an important role in the efficiency of the complex formation. The difficulties of predicting which combinations will work means that many possible combinations of the fragments need to be tested before finding an optimal pair (Fig. 2). For the AQP0‐CaM BiFC complex, their interaction is localized to the C‐terminus of AQP0. Our constructs were therefore designed with the YFP fragment fused to the N‐terminus as this would leave the C‐terminus available for the CaM interaction. It is therefore intriguing to note that the YFP_N_‐CaM + YFP_C_‐AQP0 combination used in this study was incapable of forming a fluorescent complex [Fig. 4(A)] whereas the YFP_N_‐AQP0 + YFP_C_‐CaM combination succeeded. The negative result is presumably not because of the failure of AQP0 and CaM to naturally form a complex, but instead more subtle issues may be at play. In particular, YFP is divided into fragments of 154 and 85 residues, respectively, and both are connected to the N‐termini of their fusion partners. The length of these connections can create steric restrictions as the complementary halves of YFP are brought together as the complex forms. We therefore hypothesize that the geometrical constraints imposed by the complex allow some fusions to align the YFP fragments in a manner that facilitates their combination, whereas others lead to a steric hindrance that can inhibit the YFP assembly. It is obviously difficult to judge in advance which combination may be best, and thus screening for the length of the linker may be one important variable in developing any project further. In addition, construct design is an important aspect for the purification of a homogenous product. To assure purification of the AQP0‐CaM complex, the BiFC pair (YFP_N_‐AQP0 + YFP_C_‐CaM) was recloned and the 6x‐His tag was moved to the YFP_C_ part of CaM, which allowed us to exclude the possibility that hAQP0 oligomers were co‐purified in the final product.

It is also important to appreciate that the construct design may need to be tweaked for crystallization studies. In the YFP_N_‐AQP0 + YFP_C_‐CaM that was successfully produced as a PPI complex in *S. cerevisiae*, all AQP0 monomers will be linked to a YFP_N_ fragment. Low‐resolution electron microscopy studies of the AQP0‐CaM complex^15^ indicate that two CaM molecules bind to the AQP0 tetramer, implying that only two of the four YFP_N_ fragments that are bound to the AQP0 tetramer will be occupied through an interaction with YFP_C_‐CaM. It is therefore reasonable to expect that the unbound YFP_N_ fragments may be highly flexible and might hinder the process of crystallization. This potential problem may be countered by creating constructs with additional monomer linkers, such as an YFP_N_‐AQP0‐AQP0 construct, and thereby design the BiFC study with the optimal stoichiometry for crystallization from the outset.

### Fluorescence proteins may aid crystallization

This work presents a generic BiFC‐based screening and purification protocol for recovering a stable PPI of choice. For subsequent crystal growth, the fusion partner YFP would increase the soluble fraction of the membrane protein and may help with the formation of vital intermolecular interactions as the crystal grows. This potential benefit of BiFC for crystallization has been demonstrated using split green fluorescent protein (GFP)^42^ whereby GFP β‐strands 10 and 11 were inserted into a surface loop of a target protein. Complementation assays were performed by mixing with a truncated GFP (containing strands 1–9) and observing fluorescence build up over time. GFP contributed to an ordered packing in all three dimensions and the structure of the target protein was essentially identical to its native structure. Moreover, for protein targets for which there is no prior structural information available, the fluorescent protein could also assist in solving the phases by providing a partial molecular replacement model.^42^ These speculative advantages are offset by the potential disadvantage of introducing a linker to the YFP fragments, which implies some flexibility for the YFP component of the PPI complex. An obvious trade‐off is therefore to screen the length and sequence of the linkers, which may need to be engineered in advance to optimize stability for crystallization studies. Another approach would be to raise nanobodies^43^ against the PPI complex as a whole, which bind to the complex and reduce its flexibility and thereby assist in achieving well‐diffracting crystals. The underlying rational behind the BiFC approach to PPI complex production ensures that any crystals that grow which show fluorescence will contain the correctly formed complex. This will help avoid a time‐consuming risks of dead‐ends associated with more conventional approaches to the crystallization of PPI complexes, whereby two purified proteins are mixed during crystallization trials but only one crystalizes in isolation. Alternatively, one may avoid crystallization altogether and turn to single‐particle electron microscopy for structural studies of membrane PPIs.^36^, ^37^ This elegant technique has in practice a minimum size for the complex of study which is approximately 200 MDa. Thus the addition of mature YFP to the PPI complex, which increases its overall size, is also a potential advantage of the BiFC technique. We conclude that the methodical advances for membrane protein complexes presented in this study help to lay technical foundations for the structural characterization of novel PPI targets. We believe that the elucidation of PPIs at high resolution is expected to be one of the new frontiers in structural biology as future successes will offer tremendous new biological insight.

## Material and Methods

### Genes, vectors, and strains

The AQP0 gene codon optimized for production in yeast was ordered from Genscript (Piscataway, NJ), the AQP1, AQP2, and AQP6 genes have been described previously.^33^ Protein was produced in *S. cerevisiae* (*MAT*α *ura*31 *his*311/15 *can*1100) from the p423GPD and p426GPD^44^ vectors, respectively.

### Cloning

The N‐ (YFP_N_) and C‐terminal (YFP_C_) YFP fragments consisting of amino acids 1 to 154 and 155 to 239 of the SYFP2 gene, respectively, were linked to the N‐terminus of the targets using the linker sequence KQKVMNH.^28^ The YFP fragments were cloned into the p423GPD and p426GPD vectors, respectively, using the *BamH*I and *Sal*I restriction sites. In addition, an *Afl*II site was introduced downstream of these sites for cloning of AQP0, AQP1, AQP2, AQP6, and CaM. For detection of the AQP0, AQP1 and AQP6 constructs, a hexa‐histidine tag was incorporated in the N‐terminus of the YFP fragment. For the CaM construct used in combination with AQP1 and AQP6, respectively, a C‐terminal strep tag is present (indicated in the construct name as CaM‐s in Fig. 5). For purification of the AQP0‐CaM complex, the BiFC pair was recloned and the hexa‐histidine tag was moved from the AQP0 moiety to the N‐terminus of the of the YFP_C_‐CaM moiety. For clarity reason, this version of the complex is marked with * throughout the text (YFP_N_‐AQP0 + YFP_C_‐CaM*). The truncated AQP0 construct (AQP0Δ), lacking the AQP0 C‐terminus, was generated by amplifying the YFP(_N_/_C_)‐AQP0 fusion using an internal reverse primer that introduced a stop codon at residue 221. The truncated AQP1 construct (_ΔN_‐AQP1), lacking the AQP1 N‐terminus, was generated using a primer substituting residues 1 to 9 for an *Afl*II site and subsequently cloned into the vectors containing the YFP fragments. All constructs were confirmed by sequencing (GATC‐Biotech, Germany). For chemical transformation to *S. cerevisiae*, cells (500 mL) were grown to OD = 0.7 to 1.0 in YPD medium. After harvest, cells were washed in 25 mL cold mQ followed by a wash in 1 mL 100 m*M* LiAc. Cells were resuspended in 400 µL 100 m*M* LiAc and divided into 50 µL aliquots. For the transformation, the cells were mixed with 240 µL PEG4000 (50%), 36 µL 1*M* LiAc, 50 µg freshly denatured salmon sperm, 1 µg of plasmid DNA and mQ to a final volume of 360 µL. The mixture was incubated at 30°C for 30 min followed by a heat shock at 42°C for 25 min. The cells were spun down (6000*g*, 15 s) and resuspended in 200 μL mQ before plating on selective SC‐agar plates.

### Fluorescence microscopy and image analysis

Transformants, 3‐days old, were used to inoculate 5 mL selective SC‐medium and grown at 30°C for 22 h. All images were generated using 100 ms exposure time at a Carl Zeiss axiovert 200M wide‐field fluorescence microscope fitted with a plan‐apochromat 40×/1.3 oil DIC objective. Both bright field and fluorescence images were collected. YFP was excited using 508 nm, with the light being filtered through a 500/25 band pass filter while the emitted fluorescence was filtered through a 535/30 BP filter. Images were collected using Zen blue software (Zeiss) and cell counting was performed using the ImageJ (NIH) software. For each sample, a minimum of 300 cells were counted and the fraction of fluorescent cells was calculated. To avoid bias all images were randomized and counted twice by two independent persons.

### Preparation of the YFP complexes

All cultivations (YFP_N_‐AQP0 + YFP_C_‐AQP2, YFP_N_‐AQP1 + YFP_C_‐CaM‐s and YFP_N_‐AQP0 + YFP_C_‐CaM*) were done in selective SC medium. Cells from fresh re‐streaks on agar plates were used to inoculate 5 mL medium and grown over night at 30°C. The cells were diluted 40× into 3 × 200 mL cultures and grown for 8 h for inoculation of a 30 L fermentor containing 15 L medium. The culture was grown at 14 L air/min and 800 rpm agitation. The cells were harvested in stationary phase by centrifugation (12,000*g*, 20 min) and stored at −20°C until further use.

For the AQP0‐AQP2 and the AQP0‐CaM (YFP_N_‐AQP0 + YFP_C_‐AQP2 and YFP_N_‐AQP0 + YFP_C_‐CaM*, respectively) complexes, 50 g cells were thawed and resuspended in breaking buffer (50 m*M* phosphate buffer pH 7.5, 5% glycerol) to a final volume of about 200 mL and broken by 12 cycles in the bead beater (30 s on, 90 s off). The resulting cell lysate was centrifuged (15,000*g*, 30 min) and the supernatant was spun further (158,000*g*, 90 min) to harvest the membranes. The membranes were resuspended in resuspension buffer A (20 m*M* Hepes, pH 7.8, 50 m*M* NaCl, 10% glycerol) and solubilized for 3 h at 8°C by the addition of the same volume resuspension buffer containing 4% DDM. Unsolubilized material was removed by centrifugation (140,000*g*, 30 min). A HisTrap HP column (GE healthcare) was equilibrated with Buffer A (20 m*M* Hepes, pH 7.8, 300 m*M* NaCl, 10% glycerol, 2 m*M* β‐merkapto‐ethanol, 5 m*M* CaCl_2_, 0.02% DDM) and the supernatant was loaded by continuous flow over night. Unbound proteins were washed away with Buffer A until the baseline was stable. The AQP0‐AQP2 complex was eluted by a 20 CV gradient with a final imidazole concentration of 500 mM. For the YFP_N_‐AQP0‐YFP_C_‐CaM* complex, the column was washed with 50 m*M* imidazole for 10 CV then the protein complex was eluted with 250 m*M* imidazole. Fractions showing fluorescence were pooled and concentrated in a 100,000 MWCO concentrator tube (Vivaspin). The final protein concentration for the AQP0‐CaM complex was determined with DC™ Protein Assay (Bio‐Rad) using bovine serum albumin as standard.

For the AQP1‐CaM complex (YFP_N_‐AQP1 + YFP_C_‐CaM‐s), breaking of the cells and membrane harvesting was done as for the AQP0‐CaM complex (described above). The final membranes were resuspended in resuspension buffer B (20 m*M* Tris, pH 7.0, 20 m*M* NaCl, 5 m*M* CaCl_2_, 1× Halt Protease Inhibitor Cocktail (Thermo Scientific)) and solubilized for 3 h at 8°C by the addition of the same volume resuspension buffer containing 10% β‐OG. Unsolubilized material was removed by centrifugation (257,000*g*, 30 min). The supernatant was loaded onto a Resource Q column (GE healthcare) equilibrated with Buffer A (20 m*M* Tris, pH 7.0, 5 m*M* CaCl_2_, 1% β‐OG). Unbound proteins were washed away with 2 CV Buffer A. The AQP1‐CaM complex was eluted by a 20 CV gradient with a final NaCl concentration of 1*M*. Fractions showing fluorescence were pooled and concentrated in a 100,000 MWCO concentrator tube (Vivaspin). The AQP0‐CaM and the AQP1‐CaM complexes were further purified by SEC using a Superose6 Increase column. In each case, the protein was loaded onto the column equilibrated with SEC buffer (20 m*M* Tris‐HCl pH 7.5, 100 m*M* NaCl, 5 m*M* CaCl_2_, 1% β‐OG). A calibration of the column was done in a separate experiment, where a protein mixture was run in order to establish the relationship between protein size and elution volume.

### BlueNative‐PAGE, SDS‐PAGE, immunoblot, and in‐gel fluorescence

Native‐PAGE was run according to protocol on 4 to 16% Novex Bis‐Tris gels (Invitrogen) and stained with Coomassie R‐250. The proteins were blotted onto prepared PVDF membranes for 5 h at 30 V (48 m*M* Tris, 39 m*M* glycine, pH 9.1) and then the membrane was fixed in solution A (40% methanol, 10% acetic acid). Electrophoresis under denaturing conditions was performed using NuPAGE 4–12% Bis‐tris gels (Thermofischer). Blotting was performed to nitrocellulose membranes (Hybond‐ECL, GE Healthcare) by 100 V for 1 h. For immuno detection, anti‐his6 (Clonetech), anti‐Calmodulin 2 (Proteintech), anti‐AQP2 (sc‐28629, Santa Cruz Biotechnology), and anti‐AQP0 (abcam) antibodies were used, respectively, and the chemiluminecence mode of the LAS‐1000 imaging system (Fujifilm) or Chemidoc MP molecular imager (Bio‐Rad). Images were captured with Image Reader LAS‐1000 Pro v.2.6 and analyzed using Multi Gauge V3.0 Software (both from Fujifilm) and the Image Lab™ software (Bio‐Rad). For in‐gel fluorescence electrophoresis was performed under denaturing conditions on Mini‐Protean TGX gels according to protocol (Bio‐Rad). Fluorescence was detected with Chemidoc MP molecular imager (Bio‐Rad) with excitation at 470 nm and emission at 530 nm respectively.

## Supporting information

Supporting Information Figure 1.
