# Supplementary material for: Applying bimolecular fluorescence complementation to screen and purify aquaporin protein:protein complexes
Source: Protein Sci. 2016 Sep 26;25(12):2196–208. doi: 10.1002/pro.3046 (PMC5119558; doi:10.1002/pro.3046)
Supplement: Supplementary file 1 — Supporting Information Figure 1. [file PRO-25-2196-s001.pdf]

SUPPLEMENTARY MATERIAL

Figure S1. The AQP0-CaM complex dissociates during size exclusion chromatography. Purified AQP0 and CaM were mixed in the presence of 5 mM $CaCl_2$ and run on a Superdex200 Increase column (GE-healthcare). The affinity of the AQP0-CaM complex is too weak to keep the complex intact during SEC. Two protein peaks corresponding to AQP0 (*) and CaM (**) (confirmed by SDS-PAGE and immunoblot) are eluted separately.

.

t

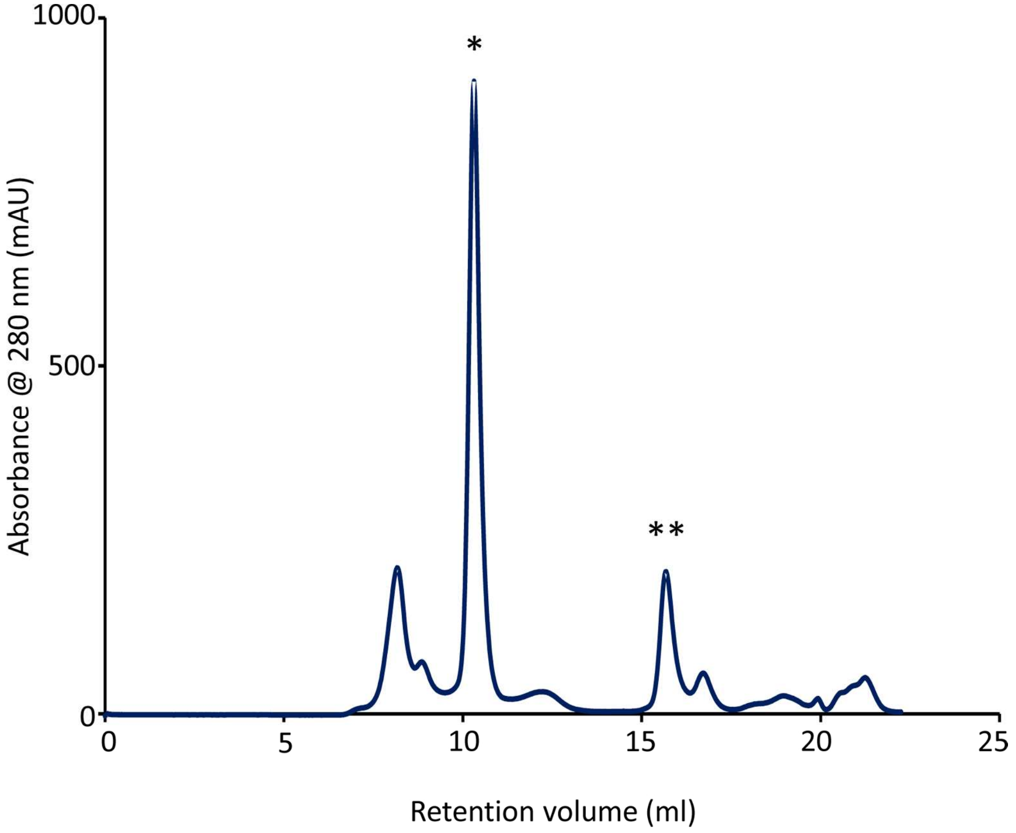

156x128mm (300 x 300 DPI)
